# O.3.3-1 “Friuli Venezia Giulia in Movimento, 10000 passi di salute” as an example of a multilevel-multifaceted project promoting citizens’ physical activity and healthy habits

**DOI:** 10.1093/eurpub/ckad133.156

**Published:** 2023-09-11

**Authors:** Laura Pagani, Gianna Zamaro, Luana Sandrin, Tiziana Del Fabbro, Alessia Del Bianco Rizzardo

**Affiliations:** University of Udine, Italy; Health Directorate, Autonomous Region Friuli Venezia Giulia, Italy; Health Directorate, Autonomous Region Friuli Venezia Giulia, Italy; Federsanità ANCI FVG, Italy; PromoTurismoFVG, Accessible and Sustainable Tourism, Autonomous Region Friuli Venezia Giulia, Italy

## Abstract

The project “Friuli Venezia Giulia in Movimento, 10000 passi di salute” (Friuli Venezia Giulia (FVG) Region on the move, 10000 healthy steps), started in 2019 and is carried out by Federsanità ANCI FVG with financial support of the FVG Region. The project’s main objective is that of encouraging physical activity and healthy habits, in an accessible way for all citizens (with a focus on the elderly) by financing the promotion of pedestrian paths located in the municipalities that have decided to pursue the initiative. The innovative aspect of the project is its multilevel-multifaceted characteristic. The project can be said to be multilevel because it involves subjects and/or institutions at different levels (region, municipalities, citizens’ associations, citizens) and multifaceted as it regards health as well as socialization, tourism, environment, and community networks.

In the early phases of the project, a participatory methodology is applied, which involves different actors through the understanding of the various needs and suggestions. A call for proposals, devoted to all the Municipalities is then open and all the applications are evaluated to detect which are accepted for funding. All the pedestrian paths funded have signals and posters with the same layout, creating a “brand” of the project. Special events are organized to open the paths, targeting all the community members, with the participation of the project’s organizing committee. News about the project and related initiatives are disseminated by social and traditional media; a dedicated website, an information line and promotional materials are created. To evaluate the project different tools are used: questionnaires, interviews, secondary data and survey sheets.

The project can be summarized with three terms: participatory, inclusive and sustainable. Due to the successful participation in the project, two spin-offs are born: walking leader courses and soft gymnastic activities for the elderly. Moreover, it has been included in a European project and has aroused the interest of other Italian regions keen on applying it to their territories. To date about 1/3 of the municipalities are involved with a potential impact on 43% of the population and 59 paths have been completed for a total of 330 km.

